# Experiences of ward rounds among in-patients on an acute mental health ward: a qualitative exploration^†^

**DOI:** 10.1192/pb.bp.113.046409

**Published:** 2015-10

**Authors:** Reed Cappleman, Zandra Bamford, Clare Dixon, Hayley Thomas

**Affiliations:** 1 South West Yorkshire Partnership NHS Foundation Trust, Wakefield; 2 Royal Bolton Hospital NHS Foundation Trust, Farnworth; 3 Division of Health Research, Lancaster University, Lancaster

## Abstract

**Aims and method** To address the gap in qualitative research examining patients' experiences of ward rounds. In-depth interviews were conducted with five in-patients on an acute mental health ward. Data were analysed using thematic analysis.

**Results** Data were organised into three first-order themes, positioned within an overarching theme relating to patients' perceptions of the use of power and control within ward rounds.

**Clinical implications** Systemic factors may make it difficult to facilitate ward rounds in a manner which leaves patients feeling fully empowered or in control, but there are practical measures to address these issues, drawn from participants' accounts.

Ward rounds are seen as a key component of care provision in in-patient psychiatric settings.^1^ However, literature in this area has found that they evoke anxiety in a large proportion of patients,^2,3^ particularly when more people are present.^4^ Patients report not feeling listened to and feeling that information is withheld from them.^3^ Many patients also feel inadequately prepared for ward rounds by staff.^5^ Labib & Brownell^3^ highlight the scarcity of qualitative investigations in this area and suggest that addressing this may highlight additional unexplored factors affecting patient satisfaction with ward rounds. This study aims to address this need. It was carried out in an acute in-patient unit located in a district general hospital in the north west of England. The need for this project to take place was identified by the unit's psychiatry team, who wished to investigate how ward rounds could be adapted to facilitate patient satisfaction. It was also hoped the project might identify avenues of investigation for future researchers, as per Labib & Brownell's suggestions.^3^

## Method

### Participants

Participants were recruited from across three mixed-gender adult acute mental health in-patient wards attached to a general hospital. Each ward has a different consultant attached. Ward rounds within this unit are chaired by psychiatrists and bring together information from members of the multidisciplinary team (nursing, psychology, occupational therapy and psychiatry), followed by a direct review of the patient's progress and mental state, formulating risk and developing treatment plan changes accordingly.

Patients from the wards were eligible to participate if they had experienced two or more ward rounds. Although nine responded favourably and completed informed consent forms, four did not participate: two were unavailable on the days they were due to be interviewed, one declined to take part when approached by the interviewer, and one chose to terminate her interview citing her mental state as non-conducive to participation. Detailed characteristics of the five participants are presented in Table 1.

**Table 1:** Participant characteristics

Patient	Gender	Age, years	Length of stay on ward, weeks	Ward rounds attended, *n*	Patient status
Patient A	Female	20	11	Too many to recall	Detained

Patient B	Male	41	4	4	Detained

Patient C	Male	36	5	4	Detained

Patient D	Male	49	2	2	Informal

Patient E	Male	27	1.5	Too many to recall	Detained

### Data collection

Ethical approval was granted from a local research ethics committee and research and development department. Participants were recruited following an initial approach being made by an assistant psychologist. All participants were interviewed in the ward's ‘quiet room’ by R.C., using an interview topic guide (questions included ‘Tell me how you've found the ward rounds here so far’ and ‘How do you feel when you're in the ward round?’). Interviews lasted between 30 and 45 min, were audio-recorded and were transcribed verbatim.

### Data analysis

Data were analysed using thematic analysis, a flexible approach for organising large amounts of qualitative data.^6^ Data analysis followed the four conventions for thematic analysis suggested by Braun & Clarke.^6^

1. Familiarisation with the data, re-reading transcripts looking for patterns and meanings.
2. Generating initial codes: organising the data into groups according to content and meaning. Transcripts were then re-coded and initial codes were grouped into ‘second-cycle’ codes.^7^ For example, initial codes from patient C's transcript, such as ‘feeling intimidated’ and ‘not being co-operative backfires’ were subsumed under the second-cycle code ‘serious consequences to not behaving in the right way’.
3. Searching for themes: second-cycle codes were grouped together into broader themes and subthemes. For example, codes such as ‘staff as a supportive network’ and ‘approval means more within a good relationship’ were grouped together in the development of a theme about the importance of relationships.
4. Reviewing themes: ensuring each theme was coherent and that themes capture the essence of the data. At this point the theme ‘power and control’ was identified as an overarching theme and remaining themes designated as first-order themes.

Coded transcripts and themes were reviewed by the research team to ensure analysis possessed sufficient quality and rigour.

## Results

As the analysis of participants' accounts progressed, data were organised into an overarching theme running throughout the data, representing the data at the highest level of abstraction, and first-level themes representing participants' accounts in less abstract, more concrete ways. The terminology of ‘overarching themes’ and ‘levels of themes’ is derived from Braun & Clarke.^6^

### First-level theme: not considering the patient's emotional state (‘They could possibly take into account a little bit more how you are at that moment in time’)

This theme concerns how the majority of participants felt that the ward round process does not take the patient's emotional state into account and actually increased their anxiety at times when it was already high. The theme's title is a quote from patient C, who reported unease at ‘probing’ questions in the ward round when he was also experiencing feelings of paranoia. Participants frequently admitted to anxiety about discussing personal issues with a group of people, some of whom were unknown to the participant:

‘Well … they can be scary at first … 'cos there's all different people there, you've got support workers, staff nurse there, there's your consultant, there's a SHO [senior house officer,] there, you can have students there, I could be there, my parents could be there. So it's like a lot of people in the ward review and, er, it's like they're all talking about you’ (patient E).

Another point raised in relation to this theme was the timing of information-giving about the ward round. For example, patient B stated that when the ward round process was initially described to him:

‘ … they use all this, all this jargon, and you know, when your head's up your arse so to speak, you don't take much of it in, you're just looking at a load of professionals and you don't know what they do.’

For patient B, information about the ward round which would have helped ameliorate anxiety was given at a time (and by a means) that did not take account of his mental state at that point.

### First-level theme: ‘behind closed doors’ (wanting more involvement in the process)

Participants felt that staff held control over ward round processes and wished for more involvement. Participants described a lack of collaboration in the area of decision-making, where they felt their views were often not taken into account and that decisions were made away from them, without their involvement.

‘ … it's like most of the things they're behind closed doors, and, and then they let you know, in your review they let you know “right we're going to follow this, we're gonna review this”.’ (patient E)

For patient A, the way in which events during ward rounds are documented was an area where control lay with staff and where she wished for more input and collaboration:

‘I think as well you should get like a copy of what they've wrote [sic] (…) 'Cos you don't know what they write down and stuff, I reckon they should tell you what they've wrote down so you could like read it for a bit and then next week feed back on what they've said and maybe like, like add things to it or develop what they've wrote.’

However, some participants devised ways of being able to have more input into the process, within the parameters they were confronted with. Patient C articulated this using a ‘game playing’ metaphor: ‘it's gotten better now because I've just got some leave you see but I wasn't entirely aware of how to play the game’. This was linked to the idea of having to adhere to unwritten rules of behaviour in the ward round: ‘there was a charge nurse in the last ward who was getting very frustrated with me because she was trying very clearly to show me the right way to behave and I was digging my heels in’. This resulted in what patient C described as a ‘meet you halfway situation, where if I cooperate with their goals they'll offer me incentives’.

### First-level theme: the importance of relationships (‘He's the only one who has listened’)

Participants stressed the importance of good relationships with staff and that such relationships had a positive impact on their ward round experiences. The theme is named after a quote from patient D, who said:

‘Like I say, he listened. That's the main thing. And when you're in. . when you're in the kind of situation I'm in at the moment, if people listen to you it's half the battle, when you've got someone you can talk to, and I felt I could talk to that doctor and he listened.’

Patient A described finding the ward rounds themselves daunting, but expressed a wish to use positive relationships she'd formed with staff to help her cope with them:

‘If you're close to that member of staff and they're sat at the side of you and if you were both speaking together … Like that would be good. 'Cos you'd feel like somebody's there for you, like, rather than being on your own.’

However, patient A also added that the parameters of these helpful relationships were controlled by staff, who may not always recognise the positive effects of staff relationships on ward round experiences. This could result in the ward round being set up in a way which does not take account of the importance of relationships for patients, for example when there are short-notice changes to which staff attend: ‘it's nerve-wracking enough going into your ward review and then at last minute, “oh yeah by the way, such and such a person isn't coming, this person's coming in”.’

### Overarching theme: power and control (‘they can keep renewing my section’)

The themes so far can be understood as part of an overarching theme relating to power and control. Issues of power and control were implicit within many issues that participants raised. The quote in this theme's name originates from patient E's interview, and relates to how some participants described their awareness during ward rounds that staff have the power to decide if they stay in hospital or leave.

Participants often talked about issues of power and control by describing staff in terms of police or other agents of the law. For example, while describing being assessed in ward rounds, patient C stated: ‘yeah, it's kind of the feeling where, I don't know if you've ever been stopped by the police but they do that kind of thing, you can feel them looking up and down at you … ’. Such comparisons seemed to arise from participants' awareness of the assessment function of ward rounds and professionals' power to determine the outcomes of these assessments. Patient B described how this awareness led to anxiety about the outcome of ward rounds: ‘ … at first, it's like having to tell these people here, if I tell them I'm having these mad thoughts, they're gonna lock me up forever’. As described in the first-level themes, participants felt that professionals hold power and control over how the ward round, and therefore the assessment process within it, is conducted. However, as indicated by the first-level theme ‘the power of relationships’, participants suggested these issues could be ameliorated by positive, collaborative relationships with staff.

Patient B also described how interpretations of the actions and intentions of those in the ward round may be influenced by previous encounters with those in authority:

‘ 'Cos my personal experience of walking into a room with loads of people is walking into a courtroom … 'Cos they sent me to jail. So, I didn't have a very good experience of loads of people if you like.’

This account suggests that some ward round procedures may evoke patients' negative memories of encounters with powerful figures.

### Suggestions for improvements

Participants seemed eager to share their ideas about practical improvements that could be made to ward rounds (Box 1). Indeed, in discussion with R.C. during recruitment, participants often cited the desire to share such ideas as their primary motivation for taking part.

**Box 1** Participants' suggestions for improvements to ward rounds

- Allow patients access to ward round records and the power to negotiate additions to them
- Invite a smaller number of staff into patients' initial ward rounds and increase the number gradually
- Be open about when patients are being assessed on particular areas of their mental state and why
- Utilise patients' one-to-one time with named nurses so ward rounds can be prepared for
- Issue patients with a booklet about hospital procedures on admission, including information about ward rounds. This would serve as an aide memoire for patients to return to so they can remind themselves of ward round procedures

## Discussion

Our participants' accounts lend support to past research indicating that patients may find ward rounds anxiety provoking.^3,5^ In support of previous findings linking ward round size to patient anxiety,^4,8^ participants in this study also spoke of the difficulties in talking to a room containing a large number of people unknown to them. Participants also reported that ward rounds are more distressing if they are already in an anxious or distressed state, an association which has not yet been studied in the quantitative literature. Findings from this study suggest that a lack of well-timed information about ward rounds could also contribute to anxiety.

As hoped, using qualitative methods led to a deeper understanding of participants' ward round experiences than has previously been possible using quantitative methods. It was hoped that more participants would be recruited but this proved difficult within the time available, owing to potential participants' apparent fluctuating mental state and their availability and motivation to take part. Although this is an exploratory study with a small sample, the findings highlight previously unexplored issues that may deserve further investigation. One such area is that of the potential importance to patients of their relationships with professionals and how sensitive use of positive relationships may positively affect the ward round experience. For example, future research might investigate whether anxiety in ward rounds is mitigated by the presence of patients' favoured members of staff, such as named nurses or key-workers.

### Improving the patient experience

Study results and participants' suggestions for improvements to ward rounds were fed back to the research site's consultant psychiatrists. This generated discussion around how the practical measures suggested by participants (Box 1) may provide safeguards to minimise the issues of power and control that inevitably influence in-patient settings, and how members of other disciplines (e.g. nursing) can play a key role in ensuring that patients feel prepared for ward rounds, supporting them to manage their anxiety in the process. The study's findings contributed to a subsequent reorganisation of ward round procedures at the research site. During the feedback process, the psychiatry team emphasised that continuing cuts to National Health Service in-patient care may lead clinicians to change how they facilitate ward rounds and that keeping the patient experience in mind will be a challenging but essential task.

Using qualitative methods to investigate acute mental health in-patients' experiences of ward rounds led to a richer understanding than has previously been possible using quantitative methods. The findings suggest possible directions for future research into ward rounds and prompted clinical discussions that have informed changes to ward round practice at the research site.
